# Peripheral Nerve Dysfunction in Middle-Aged Subjects Born with Thalidomide Embryopathy

**DOI:** 10.1371/journal.pone.0152902

**Published:** 2016-04-21

**Authors:** Alessia Nicotra, Claus Newman, Martin Johnson, Oleg Eremin, Tim Friede, Omar Malik, Richard Nicholas

**Affiliations:** 1 Department of Neurosciences, Imperial College Healthcare NHS Trust, Charing Cross Hospital, Fulham Palace Road, London, W6 8RF, United Kingdom; 2 Thalidomide Trust, 1 Eaton Court Road, Colmworth Business Park, Eaton Socon, St Neots, Cambridgeshire, PE19 8ER, United Kingdom; 3 United Lincolnshire Hospitals NHS Trust, Lincoln County Hospital, Greetwell Road, Lincoln, LN2 5QY, United Kingdom; 4 Department of Medical Statistics, University Medical Center Göttingen, 37073, Göttingen, Germany; 5 Department of Medicine, Division of Experimental Medicine, Centre for Neuroscience, Wolfson Neuroscience Laboratories, Imperial College London, London, United Kingdom; University of Modena and Reggio Emilia, ITALY

## Abstract

**Background:**

Phocomelia is an extremely rare congenital malformation that emerged as one extreme of a range of defects resulting from *in utero* exposure to thalidomide. Individuals with thalidomide embryopathy (TE) have reported developing symptoms suggestive of peripheral nervous system dysfunction in the mal-developed limbs in later life.

**Methods:**

Case control study comparing TE subjects with upper limb anomalies and neuropathic symptoms with healthy controls using standard neurophysiological testing. Other causes of a peripheral neuropathy were excluded prior to assessment.

**Results:**

Clinical examination of 17 subjects with TE (aged 50.4±1.3 [mean±standard deviation] years, 10 females) and 17 controls (37.9±9.0 years; 8 females) demonstrated features of upper limb compressive neuropathy in three-quarters of subjects. Additionally there were examination findings suggestive of mild sensory neuropathy in the lower limbs (n = 1), L5 radiculopathic sensory impairment (n = 1) and cervical myelopathy (n = 1). In TE there were electrophysiological changes consistent with a median large fibre neuropathic abnormality (mean compound muscle action potential difference -6.3 mV ([-9.3, -3.3], p = 0.0002) ([95% CI], p-value)) and reduced sympathetic skin response amplitudes (-0.8 mV ([-1.5, -0.2], p = 0.0089)) in the affected upper limbs. In the lower limbs there was evidence of sural nerve dysfunction (sensory nerve action potential -5.8 μV ([-10.7, -0.8], p = 0.0232)) and impaired warm perception thresholds (+3.0°C ([0.6, 5.4], p = 0.0169)).

**Conclusions:**

We found a range of clinical features relevant to individuals with TE beyond upper limb compressive neuropathies supporting the need for a detailed neurological examination to exclude other treatable pathologies. The electrophysiological evidence of large and small fibre axonal nerve dysfunction in symptomatic and asymptomatic limbs may be a result of the original insult and merits further investigation.

## Introduction

Phocomelia is a congenital malformation in which the proximal portions of the extremities are poorly developed or absent and the hands and feet are attached directly to the trunk or by means of a poorly formed bone [1]. It is extremely rare but emerged as a devastating side effect and one of a range of limb defects in newborn children [2–5] exposed to the drug thalidomide *in utero*. In contrast to many teratogenic drug side effects there was no dose response relationship but its impact markedly time dependent acting between 20 and 36 gestational days *in utero* [6–7], however even a single tablet at the appropriate time could result in the full blown condition. This led to its withdrawal from use in humans in 1962. Thalidomide is now used only in particular conditions where its side effects are a distal, axonal, symmetrical, predominantly sensory [8], dose-dependent [9–10] peripheral neuropathy [11–12].

Individuals born with thalidomide embryopathy (TE) have reported symptoms that may be attributable to peripheral nervous system (PNS) dysfunction in the maldeveloped limbs in later life [13, 14] and it has been hypothesized that “late onset neuropathy in thalidomiders will be recognized as a sensory equivalent of post-polio syndrome” [15]. We set out to determine whether there was any evidence of peripheral nerve dysfunction in subjects with TE limb anomalies and neurological symptoms in the upper limbs. In contrast to a previous study [14] we systematically investigated large and small somatic and sympathetic nerve function not only in the symptomatic maldeveloped upper limbs, but also in the largely asymptomatic lower limbs to ascertain whether there was any electrophysiological evidence of a more generalized neuropathic dysfunction.

## Materials and Methods

### Ethics statement

This case control study was ethically approved by the East Central London Research Ethics Committee (REC: 10/H0721/61).

### Subjects

Subjects with maldeveloped upper limbs were identified by the Thalidomide Trust’s Health Link Register on the basis of their confirmed exposure to thalidomide *in utero*, upper limb anomalies, and clinical features suggestive of neuropathy in the upper limbs. Other causes of peripheral neuropathy were excluded including diabetes, vitamin B12 deficiency, vasculitis, and paraproteins. Further exclusion criteria included prior diagnosis of cervical myelopathy or major anatomical anomalies making assessment impossible and inability to complete assessment. Healthy volunteers were identified from the Neurosciences department or were carers/friends attending with the subject. All subjects gave written informed consent and examinations took place between April 2011 and October 2014.

### Clinical and neurophysiological assessment

Subjects underwent a full neurological examination (OM) and clinical neurophysiological evaluation (AN). Assessment was limited in TE individuals due to anatomical abnormalities and/or tolerance of the tests. Nerve conduction studies (NCS) were performed (Medtronic Keypoint v5.06) on the upper (median [motor and sensory], ulnar [motor and sensory] and superficial radial [sensory]) and lower limb (peroneal [motor], tibial [motor], sural [sensory] and soleus H-Reflex). Median nerve compound muscle action potential (CMAP), when obtainable, was recorded from a thenar eminence muscle. Needle Electromyography (EMG) of one upper limb, usually limited, and/or lower limb proximal and distal muscles was performed. Sympathetic skin response (SSR) was elicited by single pulse electrical stimuli from palmar and plantar sites. Thermal threshold testing (TTT: Senselab-Thermotest Modular Sensory Analyser, 25 x 50 mm thermode) to cool and warm sensation, cold and heat pain thresholds was assessed in the distal upper and lower limbs. TTT was performed using the method of limits: three successive stimuli were given decreasing or increasing from a starting temperature of 32°C at a rate of 1°C/second. Subjects pressed a button or indicated to the examiner when the specific modality was perceived. Any delay <1 second was considered not to affect the measure of threshold as the rate of temperature change of the thermal probe was 1°C/ second.

### Statistical analysis

In NCS, TTT and SSRs we averaged the measurements of right and left limbs in each subject, where both results were available, and, if only one measure was available, that was used. NCS, TTT and SSR values, expressed as mean±standard deviation (SD), were compared between subjects and controls in Gaussian linear models adjusting for age and gender. Group differences are reported with 95% confidence intervals and p-values of t-tests in the described linear models testing the null hypothesis of no group difference. Because of the exploratory nature of the study no adjustments for multiple testing were carried out. All analyses were carried out using SAS version 9.4. A sample size of 17 patients per group yields a power of at least 80% comparing two independent means at a two-sided significance level of 5% as long as the true difference is in excess of one standard deviation. The sample size / power calculation was carried out using nQuery Advisor version 7.0.

## Results

### Clinical features and neurological symptoms in upper limb TE

Seventeen subjects (50.4±1.3 years; 10 females) with TE, upper limb anomalies and potentially neuropathic symptoms had upper limb abnormalities ranging from minimal change of an absent thumb and absent/or hypotrophic thenar eminence to double index, three or four digits only in the hand. Forearm anomalies varied from total absence to a partial anomaly but some subjects had a normal proximal upper limb. All subjects had normal appearing lower limbs, but two had a reduction in leg length. Upper limb sensory symptoms included: discomfort, numbness and pins and needles. Three subjects complained of numbness in their feet despite clinically normal lower limbs. Chronic pain was reported by seven subjects; in six affecting the upper limbs and one reported generalized pain. Seven subjects complained of weakness in their upper limbs despite the predominant sensory symptoms. One subject reported sweating dysfunction (hypohydrosis) in the hands and feet. Seventeen healthy volunteers (37.9±9.0 years; 8 females) had no limb abnormalities or neurological symptoms.

### Potentially treatable neurological damage is commonly associated with upper limb symptoms in TE

Clinical examination of TE subjects showed features of upper limb compressive neuropathy (median or cervical radiculopathy) in 15 of 17 individuals; further intervention was managed locally. The lower limb clinical neurological examination was entirely normal in 14 subjects despite three reporting numbness in the feet. In three, despite the lack of clinical symptoms, there was clinical evidence of changes consistent with a mild sensory neuropathy (n = 1), L5 radiculopathic sensory impairment (n = 1) and cervical myelopathy (n = 1), the latter required neurosurgical decompression.

### Large fibre neuropathic abnormalities are present in the affected upper limbs and sensory changes are present in the asymptomatic, clinically unaffected lower limbs

The CMAP of the median nerve showed no evokable response in six TE subjects and in some a response could only be obtained by stimulating at the axilla. The mean median CMAP amplitude was significantly reduced in subjects compared with controls. The ulnar CMAP amplitude and the amplitude of the median and ulnar sensory nerve action potential (SNAP) were not significantly different in subjects compared with controls (Table 1). Entrapment of median nerve at the distal segment (at the wrist) was seen in five subjects (bilaterally = 1, right = 2 and left = 2). The superficial radial SNAP could not be assessed in twelve TE subjects (due to radial aplasia) but it was not significantly different compared with controls. Sural nerve SNAPs were recordable in the lower limb and the mean amplitude was significantly reduced in subjects, compared with controls (Table 1). No entrapment mono-neuropathies were found in the lower limbs and no needle EMG abnormalities were found.

**Table 1:** Nerve conduction studies of the upper and lower limbs in controls (C) and thalidomide embryopathy (TE) subjects.

	Controls (n = 17) (mean +/- SD)	Thalidomide Embryopathy (n = 17) (mean +/- SD)	Difference TE—C* (95% CI, p-value)
**Upper Limbs**			
**Median CMAP amplitude (mV)**	10.2±3.5	2.8±2.1	-6.3 ([-9.3, -3.3], p = 0.0002)
**Ulnar CMAP amplitude (mV)**	10.4±3.0	7.7±3.9	-2.6 ([-6.2, 1.0], p = 0.1497)
**Median SNAP amplitude (μV)**	15.5±7.1	5.2±3.5	-4.8 ([-9.9, 0.2], p = 0.0593)
**Ulnar SNAP amplitude (μV)**	12.9±5.7	6.1±2.2	-3.5 ([-7.5, 0.5], p = 0.0845)
**Superficial radial SNAP amplitude (μV) [in a subset of 9 pts]**	27.2±9.9	14.6±7.3	-9.0 ([-18.8, 0.8], p = 0.0686)
**Lower limbs**			
**Peroneal CMAP amplitude (mV)**	7.4±3.9	4.3±4.1	-2.8 ([-7.1, 1.5], p = 0.1936)
**Tibial CMAP amplitude (mV)**	8.1±4.6	3.4±2.5	-1.7 ([-5.3, 1.9], p = 0.3493)
**Sural SNAP amplitude (μV)**	17.6±6.1	8.4±3.9	-5.8 ([-10.7, -0.8], p = 0.0232)
**Sural CV (m/s)**	59.4±6.0	50.7±5.0	-8.0 ([-13.9, -2.1], p = 0.0094)
**Soleus H reflex latency (msec)**	28.7±2.1	30.3±2.4	0.5 ([-1.7, 2.7], p = 0.6647)

* Group differences adjusted for age and gender and are reported with 95% confidence intervals and p-values of t-tests in the described linear models testing the null hypothesis of no group difference.

### Sympathetic skin responses were abnormal in the affected, symptomatic upper limbs whereas thermal thresholds were abnormal in asymptomatic lower limbs

The mean perception threshold for warm sensation was significantly increased in the clinically normal lower limbs in subjects compared with controls. Perception threshold for cool sensation and cold and heat pain thresholds were similar between TE subjects and controls in the upper and lower limbs. SSRs were present in the upper and lower limbs of TE subjects but the mean SSR amplitude in the palms was significantly reduced in the TE subject group, when compared with the controls. For the SSR amplitudes on the plantar surfaces, however, no differences in TE subjects compared with the controls could be detected (Table 2).

**Table 2:** Thermal thresholds and sympathetic skin responses in the upper and lower limbs in controls (C) and thalidomide embryopathy (TE) subjects.

	Controls (n = 17) (mean +/- SD)	Thalidomide Embryopathy (n = 17) (mean +/- SD)	Difference TE-C* (95% CI, p-value)
**Hands**			
**Warm perception threshold (°C)**	34.4±0.6	35.0±1.2	0.5 ([-0.4, 1.4], p = 0.3043)
**Cold perception threshold (°C)**	30.3±0.5	30.0±1.2	-0.5 ([-1.4, 0.3], p = 0.2029)
**Heat pain perception threshold (°C)**	42.0±4.2	44.1±4.1	3.0 ([-1.2, 7.1], p = 0.1533)
**Cold pain perception threshold (°C)**	19.1±6.7	17.4±5.5	-0.2 ([-6.7, 6.2], p = 0.9447)
**Sympathetic skin response (mV)**	1.8±0.7	1.1±0.5	-0.8 ([-1.5, -0.2], p = 0.0089)
**Feet**			
**Warm perception threshold (°C)**	35.9±1.2	39.1±3.1	3.0 ([0.6, 5.4], p = 0.0169)
**Cold perception threshold (°C)**	29.4±1.3	28.2±2.5	-1.6 ([-3.8, 0.6], p = 0.1365)
**Heat pain perception threshold (°C)**	43.8±3.5	43.3±9.0	2.6 ([-5.3, 10.5], p = 0.5102)
**Cold pain perception threshold (°C)**	17.1±6.7	16.9±5.9	-4.5 ([-11.4, 2.5], p = 0.1989)
**Sympathetic skin response (mV)**	0.8±0.7	0.6±0.7	-0.3 ([-1.0, 0.4], p = 0.3962)

* Group differences adjusted for age and gender and are reported with 95% confidence intervals and p-values of t-tests in the described linear models testing the null hypothesis of no group difference.

## Discussion

To the best of our knowledge, this is the first study systematically assessing the peripheral nervous system in both the clinically affected upper limb but also the lower limbs in middle-aged subjects born with TE. All had confirmed thalidomide exposure in utero, limb reduction defects ranging from thumb hypoplasia to phocomelia of the upper limbs with sensory symptoms, and most had normal appearing lower limbs.

In this group we found a range of problems that raised important clinical considerations for individuals with TE [14]. Three-quarters had features suggesting upper limb compressive neuropathy but, electrophysiological abnormalities consistent with compressive CTS were only found in 5 subjects. This discrepancy arises from the complex anatomy, making the typical electrophysiological findings of CTS unhelpful. In three subjects, cervical radiculopathy was suspected clinically, despite atypical dermatomal and myotomal anatomy, and subsequently confirmed on cervical magnetic resonance imaging. These findings support the need for a detailed examination of subjects with TE and neurological symptoms to exclude other treatable pathologies.

In the abnormal upper limbs, evidence of a motor large fibre dysfunction was documented. SSR was also reduced in the palms, consistent with additional sudomotor (efferent cholinergic fibre) dysfunction. Longitudinal shortening of the radial aspect in the upper limb and tibial aspect in the lower limb have been seen in the abnormal limb of phocomelic subjects [15]. Consistent with these observations our subjects had radial aspect abnormalities in the clinically abnormal upper limb but they did not have tibial aspect abnormalities. However, in the predominantly, grossly anatomically normal lower limbs, the mean amplitude of the sural SNAP was reduced. Furthermore, TTT showed that warm perception was significantly different in the lower limb in TE subjects. These findings together suggest axonal nerve dysfunction affecting not only sensory large fibers but also the small fibres. The exclusion of other potential causes of peripheral neuropathy increases the possibility that the electrophysiological abnormalities could have resulted from thalidomide exposure in utero. At the present time, the majority of TE subjects do not have clinical evidence (signs or symptoms) of a peripheral neuropathy affecting the lower limbs. As such, it is currently unclear whether the electrophysiological abnormalities found in the lower limbs have been present long term, whether these are progressive in nature, and whether they will prove to represent a clinically important part of the TE syndrome in future. Thalidomide exposure in adults in some work but not all has been found to cause a dose-dependent peripheral neuropathy [10, 16]. In TE the damage is markedly time dependent as opposed to being dose dependent [6–7]. As Thalidomide exposure in the TE group was *in utero*, it is possible that the neuropathic changes demonstrated here may have arisen from the embryonic pathobiological defects causing TE and are not likely to be a direct effect of thalidomide exposure. However, whether this is clinically relevant can only be determined with a follow-up study to determine whether further neuropathic symptoms develop and to look for evolution of the electrophysiological abnormalities.

## Supporting Information

S1 Protocol Evaluation of the peripheral nervous system in patients with thalidomide-induced limb malformations. (PDF)

S1 STROBE Checklist The Strobe checklist version 4. (DOCX)
