# Supplementary material for: Peripheral Nerve Dysfunction in Middle-Aged Subjects Born with Thalidomide Embryopathy
Source: PLoS One. 2016 Apr 21;11(4):e0152902. doi: 10.1371/journal.pone.0152902 (PMC4839770; doi:10.1371/journal.pone.0152902)
Supplement: S1 Protocol — (PDF) [file pone.0152902.s001.pdf]

Imperial College Healthcare NHS
NHS Trust

Thalidomide Study

EVALUATION OF THE PERIPHERAL NERVOUS SYSTEM IN PATIENTS WITH THALIDOMIDE-INDUCED LIMB MALFORMATIONS

Version 14, February 2010

MAIN SPONSOR: Imperial College Academic Health Science Centre

FUNDERS: Thalidomide Trust

STUDY COORDINATION CENTRE: **Imperial Healthcare NHS Trust**

REC reference: 10/H0721/61

Protocol authorised by:

Name & Role Date Signature

Study Management Group

Chief Investigators: Dr Omar Malik, Dr Richard Nicholas and Dr Alessia Nicotra
Co-investigators: Dr Claus Newman, Dr Richard Sills, and Professor Oleg Eremin
Statistician: Dr Richard Nicholas
Trial Management: Dr Omar Malik, Dr Richard Nicholas and Dr Alessia Nicotra

Study Coordination Centre

For general queries, supply of trial documentation, and collection of data, please contact:

Study Coordinator Dr Omar Malik
Address: West London Neurosciences Centre, Imperial College NHS HealthCare Trust, Charing Cross Hospital, London, W6 8RF
Tel: +44 (0)20 3311 1655
E-mail:Omar_Malik@imperial.nhs.uk
Fax: +44 (0)20 3311 1300

Clinical Queries

Clinical queries should be directed to Dr Omar Malik, Dr Richard Nicholas and Dr Alessia Nicotra.

Sponsor

Imperial College Healthcare NHS Trust is the main research Sponsor for this study.
For further information regarding the sponsorship conditions, please contact:

Ms Becky Ward
Research Governance Manager
Joint Research Office
1st Floor, Hammersmith House
Hammersmith Hospital
Du Cane Road
London W12 OHS
Tel: 020 8383 4952
Fax: 020 8383 4957

Funder

This study is being funded by the Thalidomide Trust.

*This protocol describes the '***EVALUATION OF THE PERIPHERAL NERVOUS SYSTEM IN PATIENTS WITH THALIDOMIDE-INDUCED LIMB MALFORMATIONS'** *study and provides information about procedures for entering participants. The protocol should not be used as a guide for the treatment of other participants; every care was taken in its drafting, but corrections or amendments may be necessary. These will be circulated to investigators in the study, but centres entering participants for the first time are advised to contact the trials centre to confirm they have the most recent version.*

PROTOCOL

CONTENTS

			Page
**Executive Summary**			5
**Lay Summary and Commentary**			7
**1.**	**Background**		
	1.1	Introduction	9
	1.2	Possible Modes of Action	10
	1.3	Thalidomide Embryopathy	10
	1.4	Health Link	12
**2.**	**Purpose of Study**		13
**3.**	**Aim of Study**		13
**4.**	**Study Protocol**		
	4.1	REC Approval	13
	4.2	Thalidomiders and Healthy Volunteers Studied	13
	4.3	Inclusion Criteria	13
	4.4	Exclusion Criteria	14
	4.5	Study Programme	14
	4.6	Methodology	14
	4.7	Adverse events	16
**5.**	**Documentation**		
	5.1	Data Recording and Analysis	18
	5.2	Presentations and Publications	18
**6.**	**Regulatory issues**		
	6.1	Ethics approval	19
	6.2	Consent	19

	6.4	Indemnity	19
	6.5	Sponsor	19
	6.6	Funding	20
	6.7	Audits and inspections	20
**7.**	**Study Management**		20
**8.**	**References**		20
**9.**	**Appendices**		
	9.1	Preliminary Invitation to Beneficiaries	21
	9.2	Information Sheet for Participants (a) for patient; (b) for volunteer)	23
	9.3	Consent Form for Participants (a) for patient; (b) for volunteer)	35
	9.4	GP Information Sheet from Thalidomide Trust	37

EXECUTIVE SUMMARY

BACKGROUND

- Thalidomide was introduced as a sedative agent in the UK in the 1950's. It was withdrawn from use in 1961 as a result of serious side-effects and congenital limb malformations, documented in babies born to mothers taking the drug during pregnancy.
- There is some evidence suggesting that the pathobiological abnormalities associated with Thalidomide use may be due to damage of the neural crest in the embryo and a peripheral neuropathy induced in adults. The mechanisms, however, have still to be precisely established.
- Characteristically, Thalidomiders have gross defects of the upper limbs (less so lower limbs) and a range of musculo-skeletal abnormalities. Thalidomide embryopathy results in well documented clinical profiles, involving various face and special sense anomalies, cranial nerve lesions, cardiovascular malformations and physiological abnormalities.
- Assessment of beneficiaries and feedback from Heath Link [Trust Helpline] has documented a major and escalating health problem, accentuated by the ageing process and resulting in increasing disability and morbidity, especially involving the musculoskeletal and peripheral nervous system.

PURPOSE OF STUDY

- The Trustees are very much aware of the substantial morbidity encountered by the beneficiaries, accentuated by the ageing process, and the very poor management of many of these problems in most of the beneficiaries in the UK. This is due to the rarity of the condition and inadequate support by the NHS due to lack of appropriate clinical experience and expertise and a very poor understanding of the likely causes of the major clinical problems experienced by the beneficiaries – pain (severe, stabbing, burning) pins and needles, numbness, joint problems and muscular disorders.

AIM OF STUDY

- The aim of this study is to characterise, using established clinical and investigational tools, any peripheral neuropathy (sensory, motor, sympathetic) in middle-aged beneficiaries.

STUDY PROTOCOL

- Twenty thalidomiders (selected by Health Link Register and having completed a computer-based health information questionnaire), and 10 healthy volunteers will be enrolled. Specific inclusion (upper limb anomalies, features of peripheral / central nervous system abnormalities) and exclusion (very gross abnormalities, unable to complete assessment) criteria have been established.
- The study will involve two out-patient sessions on the same day. During the first session a thorough clinical history and examination will be carried out. During the second session, clinical neurophysiological tests will be performed. It is anticipated that the study will be completed within a period of 12 months.
- Validated, well characterised methodology will be used – sensory, motor and reflex assessment, nerve conduction studies, sympathetic skin response and thermal threshold testing.
- Data recording will use clinical notes only and undergo statistical analysis. Data will be available to PIs (AN, RN and OM) and Co-Is (CN, RS and OE).
- On completion of the study a Report (including a Lay Summary) will be produced for presentation to, and discussion by, the Trustees and their Medical Advisors. Any presentations and publications emanating from the study will give due acknowledgement to the Trust and be seen by the Trustees prior to entering the public domain.

LAY SUMMARY AND COMMENTARY

The Trustees of the Thalidomide Trust are very much aware of the substantial and significant morbidity encountered by the thalidomiders, accentuated in an escalating manner by the ageing process, and the limited management of and support provided by the NHS in the UK. The Trustees are concerned about these medical (and psychological) problems and have empowered the Health and Welfare Group (H&WG) in the Trust to address these issues and how they may be resolved or improved.

The H&WG, with representation from the National Advisory Committee (NAC), after comprehensive discussions and with the approval of the Trustees, has established a modest Health Link infrastructure to provide information and support to those beneficiaries seeking help and guidance about their condition, and to direct and facilitate their access to those aspects of the NHS (primary and tertiary) which would provide the necessary and appropriate level of support and treatment.

An important element of the Health Link set-up is the creation of an anonymous Register, to record and collate the clinical features (and psychological concerns) experienced by the beneficiaries, defining more precisely the spectrum of disorders and their frequencies, in the beneficiaries registered with the Trust. The information gathered, to-date, has confirmed the need for such a service and highlighted the complexity of the medical problems. It has also documented the poor level of support and benefit provided for Thalidomiders by the NHS in the UK.

There are a variety of reasons for this very poor management of most beneficiaries. Firstly, the abnormalities of anatomy and tissue function found in Thalidomiders are limited to a small, albeit important, group of individuals. This rare clinical condition, and low national priority, has inevitably led to a dearth of clinical experience and expertise in the NHS. Secondly, the symptom complex in most beneficiaries is multi-factorial, very poorly understood and the contribution of different causes, including any thalidomide-induced peripheral nerve damage, is unclear and often confusing.

This failure of understanding the basic elements contributing to the clinical problems and lack of the necessary clinical expertise has contributed to the poor quality of advice provided and treatment offered to Thalidomiders.

The H&WG also established a sub-group/committee to explore the feasibility of carrying out a pilot project to study a small cohort of Thalidomiders (and healthy controls), identified with significant and relevant symptoms (pain, numbness, pins and needles, involving joint and muscles).

The purpose of this study is to define more precisely the contribution of peripheral nerve damage to the complex symptoms experienced by the beneficiaries. Hopefully, this may lead to a more specific, targeted and more beneficial therapeutic approach to be used in patient management.

We have identified a group of specialists with the necessary expertise (Dr Omar Malik, Dr Richard Nicholas and Dr Alessia Nicotra), based at the West London Neurosciences Centre, Charing Cross Hospital, London. We are fortunate in collaborating with this Centre of excellence. The study is perceived as being mutually advantageous as Drs Malik, Nicholas and Nicotra are interested in studying this area.

A formal Report, with an accompanying Lay Document, will be produced and presented to the Trustees for information, evaluation and any further action by the Trust.
Prior to any presentations or publications in the public domain the intended material will be accessible to the Trustees and their Medical Advisors for their comment and due acknowledgement will be given to the Thalidomide Trust.

1. BACKGROUND

1.1 Introduction

1.1.1 Thalidomide (alpha-phthalimido-glutarimide) is believed to have been developed in the 1940s and may have been tested in Nazi-occupied France and possibly in concentration camps in Poland (1). It was subsequently evaluated by Gruenenthal as an anticonvulsant agent, shortly after World War II. It was found to be unsuitable for this purpose but shown to have sedative properties. It was demonstrated to be an effective drug and overdoses were not lethal (2). It replaced barbiturates from 1957 (Germany) onwards, until it was withdrawn towards the end of 1961 (later in Japan). It was removed from use in the UK (marketed as Distavel) as a result of the serious side-effects (irreversible peripheral neuropathy) and devastating congenital malformations, documented in babies born to mothers taking the drug during pregnancy (2, 3).

1.1.2 Recently, it has been introduced as a treatment for certain groups of adult patients with myeloma (3). In such patients, peripheral neuropathy has been shown to occur due to damage of the mature and fully developed peripheral nervous system.

1.1.3 When assessing patients, it will be difficult to differentiate any specific thalidomide-induced neuropathy from a range of extrinsic factors and abnormalities arising as a consequence of the severely disturbed growth and anatomical anomalies eg (phocomelia) in the limbs. Abnormal stresses on normal or improperly developed joints due to repeated and long-term patterns of activity results in premature and exacerbated osteoarthritis (eg cervical spondylosis), chronic muscular strain and inflammatory changes in fibrous connective tissues and tendons.

1.1.4 Also, unrelated (non thalidomide-induced) factors such as the thoracic inlet syndrome, nerve entrapment on crossing the humerus and carpal tunnel compression, may contribute to the symptoms and defective function documented in Thalidomiders. Hence, all these various contributing factors may make it difficult to establish the presence of any intrinsic thalidomide-induced neuropathy.

1.2 Possible Modes of Action

1.2.1 McCredie in her recent book (2), suggests the drug damages the neural crest of the developing embryo, thereby, causing abnormal sclerotome developments. This postulate, supported by experimental evidence in animals, offers an explanation for many of the observed outcomes in humans. However, this theory, does not explain the apparent resistance to neural damage in the segments above and below C5, 6 and 7 in the upper part of the body. Moreover, there is no such resistance in naturally occurring limb reduction defects, as a consequence of hereditary or idiopathic causes.

1.2.2 Central nervous system and associated developmental defects of the special senses (ear, eye), as well as certain cranial nerves, may also occur. The Vth , Xth , XIth and XIIth cranial nerves appear to escape damage (Dr C Newman – personal communication, 2009). Various syndromes (Crocodile Tear, Duane and Marcus Gunn) have, in the past, been linked to thalidomide-induced damage.

1.2.3 Differences in the axiality of limb reduction defects found in sporadic or genetic causes of phocomelia, are highlighted when compared with thalidomide-induced changes. In the latter, only the pre-axial (C5, C6, C7) pattern occurs in the upper limbs. In the former, both pre-axial and post-axial patterns (C8, T1) are found. In both axial patterns, complete absence of the limbs can occur. In the post-axial pattern, high 'amputation-like' limb deficiencies may occur, above the elbow and without digit buds.

1.2.4 Autonomic nervous system dysfunction is not well studied nor well documented. Newman, in over 50 years of clinical practice and working as a Medical Advisor to the Trust, has recorded only a few examples of bladder dysfunction (personal communication, 2009). As far as we are aware, there have been no clinical and laboratory studies of bowel, bladder, sexual and vascular autonomic function carried out and the findings published in peer-reviewed journals.

1.2.5 A recently published study from Aberdeen suggested that thalidomide may also have a detrimental effect on blood vessel development by inhibiting angiogenesis.

1.3 Thalidomide Embryopathy

Clinical profiles that have been documented in Thalidomiders include:

1.3.1 ***Limb Abnormalities***: upper limb abnormalities are the commonest but both upper and lower extremities can be affected, very rarely lower limb defects alone. Preaxial reduction defects, from hypoplasia, of the thumb (or, alternatively, thumb triphalangism) to total limb absence can occur. Limb reduction defects occur bilaterally in 80 – 85% of cases, though with a variable degree of asymmetry.

a) *Hypoplasia of first and second digits* – typical abnormality with hypoplasia of supporting carpal bones and variable long bone defects (deficiency of radius), with or without deficiency of glenoid process of scapula and associated subluxation of the shoulder.

b) *Ulna shortening* - is less pronounced than that of the radius and may be the only long bone, fused to a humeral remnant and supporting the fourth and fifth digits, close to the shoulder.

c) *Phocomelia* ('seal limb') - is the term given to a very short, paddle shaped arm with two or three digits.

d) *Thumb triphalangism* – where the thumb is changed to an index finger-like digit arising next to the index, with wrist and forearm preserved. Rarely the thumb may be duplicated.

e) *Foot abnormalities* – hypoplasia of the great and second toes occur with reduction defects of the tibia, but there may be instead (quite frequently) big and second toe duplication leading to a polydactylic foot. The foot itself is usually preserved virtually intact though frequently in a club foot position.

1.3.2 **Musculo-skeletal System**: Abnormalities of muscle, connective tissues, spine and pelvis are present to a variable degree.

a) *Muscles* – may be hypoplastic, fused, absent or have abnormal insertions; function in adult thalidomiders appears normal, but strength is impaired if the muscle is under developed or works with a mechanical disadvantage from abnormal anatomy. Hypoplastic preaxial and surviving postaxial fingers (ie 4th, 5th), even if of normal length, may nevertheless show flexion deformities and limited movement, probably from tendon shortening.

b) *Spine* – abnormalities of the inter-vertebral discs and epiphyseal plates occur resulting in anterior fusion of adjacent vertebral bodies with the associated kyphosis or scoliosis. Prominent ossification of the anterior spinous ligament with loss of flexibility is

seen. In the cervical spine, especially, spondylosis of the intervertebral joints is common spondylolysis / spondylolisthesis may occur at L5. Spina bifida occulta and, less frequently, spinal canal stenosis also occur in thalidomiders.

c) *Bony pelvis* – shows varying degrees of aplasia with sacrum being malformed or partly absent, absent pubic ramus, deficient or absent hip socket.

1.4 Health Link

1.4.1 The ageing process may affect the Thalidomide Trust beneficiaries differentially, contributing to increased perceived disability and morbidity.

Through the Health Link Helpline set up by the Trust, the following have been identified:

Musculoskeletal Problems: These include shoulder, neck, hip, and back

Pain: Chronic, with an unidentified source

Pins and Needles: As shown by the questionnaire statistics below. This is a very common symptom amongst the beneficiaries.

Abnormal Sweating: This is beginning to show up as quite a common symptom. However, it was commonly reported in childhood, and 'explained' (with little evidence) on the basis of reduction of surface area available for cooling.

Accidents: Are becoming a common problem, particularly because of a lack of understanding by Health Care workers that if already disabled a fairly minor loss of function can destabilise a situation and render the individual much more severely disabled. Unfortunately, we have had a couple of instances of "accidents" during medical or surgical treatment.

1.4.3 In 2008, a postal survey with 175 responders self-reported neuropathic symptoms (pain, numbness, tingling) to be a problem in 75% of cases.

2. PURPOSE OF THE STUDY

The purpose of the study, therefore, is to define more precisely the contribution of peripheral nerve damage, irrespective of the cause, experienced by the Thalidomiders.

3. AIM OF STUDY

To characterise, using established clinical and investigational tools, any peripheral neuropathy (sensory, motor and sympathetic) in a cohort of middle-aged Thalidomiders.

4. STUDY PROTOCOL

4.1 REC Approval

Clinical study and patient enrolment will only commence when approval has been given by the Research Ethics Committee.

4.2 Thalidomiders and Healthy Volunteers Studied

Twenty thalidomiders will be enrolled into the study. Cohort of patients will be screened and selected for possible participation from the Health Link Register. The Thalidomide Trust will contact the thalidomiders' GP/PCT and will ask for a referral for them to be seen at Charing Cross. Once the referral is received by the PIs of the study, the thalidomiders will be invited to attend their study visit at Charing Cross Hospital.

Ten healthy volunteers (carers or friends of thalidomiders accompanying them) will also be invited to take part in the study.

4.3 Inclusion Criteria

4.3.1 Patients identified from the Register will be invited to participate in the clinical study. Healthy volunteers, aged between18 to 65 inclusive and with no neurological or musculo-skeletal disorders, will also be invited to participate.

4.3.2 Patients and healthy volunteers will have read and understood the Patient and Healthy Volunteer Information Sheets, respectively, and signed the appropriate Informed Consent forms.

4.3.3 All thalidomiders enrolled will have upper limb anomalies and clinical features suggestive of peripheral and / or central nervous system abnormalities.

4.3.4 All invitees will be able to undergo the neurological examination, nerve conduction studies, sympathetic skin responses and thermal threshold testing, albeit limitations may be imposed by anatomical abnormalities.

4.4 Exclusion Criteria

4.4.1 Patients, or healthy volunteers, unable or unwilling to sign the Informed Consent.

4.4.2 Thalidomiders with very gross abnormalities and unlikely to complete the neurological and investigation assessments. Healthy volunteers unwilling to comply with the study protocol.

4.4.3 Patients who do not have unequivocal thalidomide –induced anomalies and nervous system symptoms.

4.4.4 Individuals with hereditary congenital limb reduction and related abnormalities.

4.4.5 Women of child-bearing potential who are pregnant.

4.5 Study Programme

4.5.1 The pilot study will consist of two out-patient sessions on the same day. During the first session thalidomiders will have a clinical history taken and a clinical examination. In the second session, a clinical neurophysiological evaluation will be carried out: this will last 2-3 hours.

4.5.2 Healthy subjects will be evaluated during a single session where they will undergo neurophysiological assessments.

4.6 Methodology

4.6.1 *Sensory Examination*

Dermatomal light touch and pin- prick on a 2 point scale as follows: normal=2, impaired=1, absent=0.

Referred pain will be assessed on a Visual Analogue Scale: 0=no pain, 10=worst pain.

4.6.2 *Motor Examination*

Muscle strength of upper limb (elbow flexors, wrist extensors, elbow extensors, finger flexors, finger abductors) and lower limb (hip flexors, knee extensors, ankle dorsiflexors, long toe extensors, ankle plantar flexors) will be assessed and key muscles will be scored on a 5 point grading scale as shown below:

5 = active movement, full range of motion, against gravity and provides full resistance

4 = active movement, full range of motion, against gravity and provides some resistance

3 = active movement, full range of motion, against gravity

2 = active movement, full range of motion, gravity eliminated

1 = palpable or visible contraction

0 = total paralysis

Thalidomiders will have limitations (variable degree) with performing this test due to their musculo-skeletal variants; these limitations will be recorded in the Clinical Report Form and will be taken into account when analysing data.

4.6.3 *Reflexes*

Biceps, triceps, knee, and ankle reflexes as follows: 2=normal, 1=diminished, 0=absent.

4.6.4 *Nerve Conduction Studies*

Nerve conduction studies (NCS) will be carried out using a Medtronic EMG machine using surface recording and stimulating electrodes and will include the following:

(a) Upper limb - median (sensory and motor), ulnar (sensory and motor), superficial radial (sensory).

(b) Lower limb - peroneal (motor), tibial (motor), sural (sensory), soleus H Reflex.

The NCS will take into account the patient's anatomy (normal, deranged), in the upper and/or lower limbs.

4.6.5 *Sympathetic Skin Response*

The sympathetic skin response (SSR) will be carried out with a Medtronic EMG machine using surface electrodes. The recording electrodes will be applied to palmar and/or plantar sites taking into account the patient's anatomy (normal, deranged). The SSR will be evoked by a single pulse electrical stimulus.

4.6.6 *Thermal Threshold Testing*

Thermal threshold to cool sensation, warm sensation, cold pain and heat pain will be measured with a SENSELab-THERMOTEST Modular Sensory Analyser; a thermode (25 x 50 mm) will be applied to the skin to be tested in the distal part of the upper and / or lower limbs. Thermal sensory thresholds will be measured using the method of limits: subjects will receive successive, decreasing or increasing, thermal stimuli from a starting temperature and will be required to arrest the changing stimulus intensity by pressing a button as soon as they perceive the specific modality being tested.

4.6.7 *Electromyography (EMG) Examination*

Needle electromyography will be carried out using a Medtronic EMG machine and a standard needle recording electrode. At least one upper limb and/or lower limb proximal and distal muscles will be examined to look for possible neuropathic or myopathic changes.

4.7 ADVERSE EVENTS

4.7.1 DEFINITIONS

Adverse Event (AE): any untoward medical occurrence in a patient or clinical study subject.

Serious Adverse Event (SAE): any untoward and unexpected medical occurrence or effect that:

· **Results in death**

· **Is life-threatening –** *refers to an event in which the subject was at risk of death at the time of the event; it does not refer to an event which hypothetically might have caused death if it were more severe*

· **Requires hospitalisation, or prolongation of existing inpatients' hospitalisation**

· **Results in persistent or significant disability or incapacity**

· **Is a congenital anomaly or birth defect**

Medical judgement should be exercised in deciding whether an AE is serious in other situations. Important AEs that are not immediately life-threatening or do not result in death or hospitalisation but may jeopardise the subject or may require intervention to prevent one of the other outcomes listed in the definition above, should also be considered serious.

4.7.2 REPORTING PROCEDURES

All adverse events should be reported. Depending on the nature of the event the reporting procedures below should be followed. Any questions concerning adverse event reporting should be directed to the Chief Investigator in the first instance.

4.7.3 Non serious AEs

All such events, whether expected or not, should be recorded.

4.7.4 Serious AEs

An SAE form should be completed and faxed to the Chief Investigator within 24 hours.

All SAEs should be reported to the <name of REC> where in the opinion of the Chief Investigator,the event was:

· 'related', ie resulted from the administration of any of the research procedures; and

· 'unexpected', ie an event that is not listed in the protocol as an expected occurrence

Reports of related and unexpected SAEs should be submitted within 15 days of the Chief Investigator becoming aware of the event, using the NRES SAE form for non-IMP studies. Local investigators should report any SAEs as required by their Local Research Ethics Committee, Sponsor and/or Research & Development Office.

Contact details for reporting SAEs

Fax: 020 3311 1300

Please send SAE forms to: Dr Omar Malik

Tel: 020 3311 1655 (Mon to Fri 09.00 – 17.00)

5.0 DOCUMENTATION

5.1 Data Recording and Analysis

5.1.1 ***Clinical and Investigational Data***

Clnical Data will be recorded in the patient's clinical notes. The clinical neuropathophysiological data generated will be stored in the data acquisition computer and then analysed off-line. Data will undergo statistical analysis.

5.1.2 ***Data Protection***

Patient data will be protected by the usual data protection protocols. Anynomization will start at study entry and maintained since.

Data will be kept in a dedicated and safe area of storage.

5.1.3 Data will be available to the Principal Investigators (PIs) and the Co-investigators (Co-Is). This may be accessed for preliminary analysis and / or following completion of the study and the production of a formal Report.

5.2 Presentations and Publications

5.2.1 ***Study Report***

Following completion of the pilot study a Report will be produced documenting the data obtained, highlighting any key and / or unanticipated findings and setting out recommendations for possible further studies and / or guidelines for patient management, if feasible. The Report will have an accompanying lay summary. The Report will be presented to the Trustees and their Medical Advisors.

5.2.2 ***Agreement***

Any presentations at meetings and any submissions for publication in peer-reviewed journals, will have variable contributions by all investigators but the two PIs (AN, RN and OM) will be the main authors and the Co-Is (RS, CN, OE) as named co-authors. The contents and conclusions will be scrutinised and agreed by all concerned before entering the public arena.

There will be a grant totaling £12,000 provided by the Thalidomide Trust to cover ALL expenses incurred, including clinician time, facility fee, consumables, cost of hiring of equipment and administration support. This grant will cover the cost of all 30 subjects (20 patients and 10 healthy volunteers/controls).

5.2.3 **Acknowledgement**

In all presentations and publications due acknowledgement will be made to the Thalidomide Trust, and will be seen by the Trustees and their Medical Advisors prior to entering the public domain.

6. REGULATORY ISSUES

6.1 Ethics approval

The Study Coordination Centre has obtained approval from the East Central London Research Ethics Committee (REC) 1.

The study must be submitted for Site Specific Assessment (SSA) at each participating NHS Trust. The Study Coordination Centre will require a copy of the Trust R&D approval letter before accepting participants into the study. The study will be conducted in accordance with the recommendations for physicians involved in research on human subjects adopted by the 18th World Medical Assembly, Helsinki 1964 and later revisions.

6.2 Consent

Consent to enter the study must be sought from each participant only after a full explanation has been given, an information leaflet offered and time allowed for consideration. Signed participant consent should be obtained. The right of the participant to refuse to participate without giving reasons must be respected. After the participant has entered the trial the clinician remains free to give alternative treatment to that specified in the protocol at any stage if he/she feels it is in the participant's best interest, but the reasons for doing so should be recorded. In these cases the participants remain within the study for the purposes of follow-up and data analysis. All participants are free to withdraw at any time from the protocol treatment without giving reasons and without prejudicing further treatment.

6.3 Confidentiality

Participants' identification data will be required for the registration process. The Study Coordination Centre will preserve the confidentiality of participants taking part in the study and is registered under the Data Protection Act.

6.4 Indemnity

Imperial College London holds negligent harm and non-negligent harm insurance policies which apply to this study.

6.5 Sponsor

Imperial College Academic Health Science Centre will act as the main Sponsor for this study. Delegated responsibilities will be assigned to the NHS trusts taking part in this study.

6.6 Funding

The Thalidomide Trust is funding this study.

6.7 Audits and inspections

The study may be subject to inspection and audit by Imperial College Academic Health Science Centre under their remit as Sponsor, the Study Coordination Centre and other regulatory bodies to ensure adherence to GCP.

7. STUDY MANAGEMENT

The day-to-day management of the trial will be co-ordinated through by Dr Omar Malik, Dr Richard Nicholas and Dr Alessia Nicotra.

Study Coordination Centre. Overall study management will be undertaken by the Chief Investigator, Dr Omar Malik.

8. REFERENCES

(1) Anon, Sunday Times News 9/2/2009 p16,

(2) Smithells R W and Newman C G H, Recognition of thalidomide defects. J Med Genet, 1992; 29: 716-723

(3) McCredie, J, .Beyond Thalidomide. Birth Defects explained. R Soc Med Press, 2007

Appendix 9.1: Preliminary Invitation to Beneficiaries

The Thalidomide Trust

Registered Charity No. 266220

Trustees:
Sir Michael Wright
C.D. Lever
Professor O. Eremin
R.H. Lawson
M. Napier CBE QC
Professor C. Glendinning
J.S. Curtis
M. Winfield OBE
J.H. Howard
Professor Dame Lesley Southgate

Director:
Dr. M.W. Johnson BD PhD MSc

Registered Address:
1 Eaton Court Road
Colmworth Business Park
Eaton Socon
St Neots
Cambridgeshire
PE19 8ER

Telephone: 01480 474074
Fax: 01480 226777
E-mail: administration@thalidomidetrust.org
Web site: www.thalidomidetrust.org

Dear Beneficiary

We are very pleased to inform you that we have established contact with Consultant Neurologists, Drs Omar Malik/Richard Nicholas and their team, based at the West London Neurosciences Centre, Charing Cross Hospital, who are particularly interested in the health issues and problems that are affecting you and other Beneficiaries. We understand that you are having significant health problems with particular symptoms and that you would welcome help with these health matters. Drs Malik/Nicholas have agreed to see Beneficiaries if they are referred by their GP / PCT and we believe that a better understanding may be of benefit.

Drs Malik and Nicholas already have some experience in this area. We believe that the best approach to the health problems that are affecting you and other Beneficiaries is to build up the necessary experience in one centre, the resultant knowledge and expertise acquired can then be used in the future for other such patients.

It is entirely right and proper that investigations and treatment for your health problems should be provided by the NHS. The Thalidomide Trust is not able to pay for such treatment. However, the

Thalidomide Trust has agreed to pay for some specific tests that would not normally be done by the NHS in order to attempt to establish the possible reasons for the symptoms that you are having.

We hope that the results from your consultation may result in better treatments being offered in the future. These tests will be done as part of a clinical research study and the full criteria and arrangements for this will be given to you in other documents in due course.

As you have significant health symptoms we would encourage you to approach your GP and PCT with a view to a referral to Drs Malik/Nicholas as an NHS patient. We will also be writing to your GP asking him/her to support this request.

Yours sincerely,

On behalf of the Thalidomide Trust

Appendix 9.2 : a) Participant's Information Sheet for Patients

Imperial College Healthcare NHS
NHS Trust

West London Neurosciences Centre
Imperial College London
Charing Cross Hospital
London, W6 8RF

INFORMATION SHEET FOR PARTICIPANTS

Title

Evaluation of the peripheral nervous system in patients with thalidomide-induced limb malformations

Investigators: Dr Omar Malik, Dr Richard Nicholas, Dr Alessia Nicotra

(Information Sheet V4 – 21st December 2010)

You are being invited to take part in a research study. Before you decide it is important for you to understand why the research is being done and what it will involve. Please take time to read the following information carefully and discuss it with others if you wish.

- Part 1 tells you the purpose of this study and what will happen to you if you take part
- Part 2 gives you more detailed information about the conduct of the study

Please ask us if there in anything that is not clear or if you would like more information. Take time to decide whether or not you wish to take part. Thank you for reading this.

PART 1

What is the purpose of the study? –
The purpose of this study is to understand if some of the clinical problems you experience, like pain, pins and needles, numbness, are due to damage to the peripheral nervous system (PNS).

The PNS is a communication network made of nerves specialized in conducting information from the distant part of the body to the brain and spinal cord and from the brain and spinal cord to the distant part of the body.
Damage to the PNS is called peripheral neuropathy. This study will help us understand if people born with thalidomide-induced limb malformation have a peripheral neuropathy.

Why have I been chosen? –
We are asking you to think about joining this study because you are known to have clinical problems, which could be due to a peripheral neuropathy.
For this study, we hope to recruit at least 20 people like yourself who have the same clinical problems.

Do I have to take part?
No. It is up to you to decide whether or not to take part. If you do decide to take part you will be asked to sign a consent form. If you decide to take part you are still free to withdraw at any time and without giving a reason. A decision to withdraw at any time, or a decision not to take part, will not affect the standard of care you receive.

What will happen to me if I take part? –
You will be invited to take part by the Thalidomide Trust.
Your GP will be aware you are being invited to take part in this study. We will check if you are eligible to take part with your GP or consultant. We will discuss the study with you and answer any questions you may have.

If you decide to take part you will come to Charing Cross Hospital for a single out-patient visit during which two assessment sessions will be carried out.
During the first session, a doctor will take your clinical history and perform a thorough neurological examination. This will include testing muscle strength, tendon reflexes, your ability to feel touch and pin-prick.

During the second session you will have more specialized tests, which are carried out routinely in many people who come to our Center for a neuropathy screen.
These tests are:

1) Nerve conduction studies (NCS): it measures the degree of damage in the larger nerve fibres.
   During this test, a probe (electrode) electrically stimulates nerve fibres, which respond by generating their own electrical impulse: this is captured by another electrode placed along the nerve pathway or on the skin over a muscle.

2) Sympathetic skin response (SSR): it measures the function of sweating.
   During this test, the electrodes are placed on the skin of your hands or feet and will record the change in sweat activity following a stimulus.

3) Thermal threshold testing (TTT): it measures your ability to feel cool and warm sensation and mild pain sensation induced by cold and warmth.
   During this test, a probe will touch the skin on some part of your body (hands, feet) and you will be asked to press a button as soon as you feel cold and heat, cold pain and heat pain.

4) Electromyography (EMG): it studies the activity of muscles when they are at rest and when they contract. During this test, a fine needle is inserted into some muscles and picks up their activity.

   Insertion of a needle for the EMG test could be uncomfortable; however, if discomfort is felt, usually is mild and lasts only a couple of minutes. If you feel any unacceptable discomfort you can request that the procedure be stopped.

These special tests will complete your participation in the study.

What do I have to do? –

You will need to attend a single out-patient visit, during which two assessment sessions will be carried out, at Charing Cross Hospital.

The doctors involved with this study will carry out the assessments described above.

You are free to withdraw from the study at any time without giving a reason.

What is the drug or procedure that is being tested? –

There are no drugs being tested in this study and no new treatments.

This study is simply looking at the functioning of your peripheral nerves. All the tests, including the specialised tests, are carried out on a daily basis.

What are the side effects of any treatment received when taking part? –

There are no additional treatments being tested in this study.

What are the possible disadvantages and risks of taking part?

The assessment techniques are entirely safe and non-invasive and there are no known risks from having these test performed. Insertion of a needle for the EMG test could be uncomfortable; however, if discomfort is felt, usually is mild and lasts only a couple of minutes. If you feel any unacceptable discomfort you can request that the procedure be stopped.

All tests will be performed within your limits of tolerance.

Although pregnancy is not a contraindication, women who are pregnant will not be included.

What are the possible benefits of taking part? –

There are no clear immediate benefits of taking part. However, the information we get might help improve the future assessment and treatment of people with the same condition as yours.

What happens when the research study stops?

We will make any published results available to all participants in the study.

Contact details for further information:

If you are unsure about this study and would like to consider further before you make your decision, please take your time to do so. You may ask for further information by telephoning

The people to contact are:

Dr Omar Malik / Dr Richard Nicholas / Dr Alessia Nicotra

Charing Cross Hospital

West London Neurosciences Centre

Fulham Palace Road

London W6 8RP

Tel. 020 3311 1655

This completes Part 1 of the Information Sheet

If the information in Part 1 has interested you and you are considering participation, please continue to read the additional information in Part 2 before making any decision.

PART 2

What if relevant new information becomes available?

Sometimes during the course of a research project, new information becomes available about the technique that is being studied. If this happens, your research investigator will tell you about it and discuss whether you want to or should continue in the study. If you decide not to carry on, your research investigator will make arrangement for you care to continue. If you decide to continue in the study, you will be asked to sign an updated consent form.

What will happen if you don't want to carry on with the study?

You can withdraw from the study at anytime. If we collected data from you, we may still wish to use it in our analysis. Any information that can be clearly identified as yours can be destroyed if you wish.

What if there is a problem? –

In case something goes wrong during your participation in the above research study, you will be covered by the standard NHS Hospital Indemnity for negligent or wrongful harm by Imperial College Healthcare NHS Trust.

Will my taking part in this study be kept confidential?

All information which is collected about you during the course of the research will be kept strictly confidential. Where applicable, any information about you which leaves the hospital will have your name and address removed so that you cannot be identified from it. Any data collected will not be provided to your GP or hospital consultant without your prior consent.

What will happen to the results of the research study?

The results of the study will be presented at scientific meetings and published in scientific journals. We can make any published results available to participants in the study by sending reprints by post or email upon request. Study participants will not be identifiable in any report or publication unless the participant has been specifically consented to release such information.

Who is organising and funding the research? –

The study is organized by doctors at Charing Cross Hospital, Imperial College Healthcare Trust, London, in close collaboration with the Thalidomide Trust.

Will there be expenses incurred if I take part?

Yes, your travel expenses (and that of your accompanying person if appropriate) will be paid by the Thalidomide Trust.

Who has reviewed the study? –

This study was approved by the East Central London Research Ethics Committee (REC) 1.

Contact details for further information:

You may ask for further information by telephoning

Dr Omar Malik / Dr Richard Nicholas / Dr Alessia Nicotra

Tel. 020 3311 1655

West London Neurosciences Centres
Imperial College London
Charing Cross Hospital
London, W6 8RF

INFORMATION SHEET FOR HEALTHY PARTICIPANTS

Title

Evaluation of the peripheral nervous system in patients with thalidomide-induced limb malformations

Investigators: Dr Omar Malik, Dr Richard Nicholas, Dr Alessia Nicotra

(Information Sheet V4 – 21st Dec 2010)

You are being invited to take part in a research study. Before you decide it is important for you to understand why the research is being done and what it will involve. Please take time to read the following information carefully and discuss it with others if you wish.

- Part 1 tells you the purpose of this study and what will happen to you if you take part
- Part 2 gives you more detailed information about the conduct of the study

Please ask us if there in anything that is not clear or if you would like more information. Take time to decide whether or not you wish to take part. Thank you for reading this.

PART 1

What is the purpose of the study? –
The purpose of this study is to understand if some of the clinical problems people born with thalidomide-induced limb malformations, like pain, pins and needles, numbness, are due to damage to the peripheral nervous system (PNS).

The PNS is a communication network made of nerves specialized in conducting information from the distant part of the body to the brain and spinal cord and from the brain and spinal cord to the distant part of the body.
Damage to the PNS is called peripheral neuropathy. This study will help us understand if people born with thalidomide-induced limb malformation have a peripheral neuropathy.

Why have I been chosen? –

We are asking you to think about joining this study because we need people who do not have clinical problems of peripheral neuropathy to compare with people born with thalidomide-induced limb malformations who have such clinical problem.
You do not have clinical problems of peripheral neuropathy and you would be an ideal subject to act as a normal control for our patient group.

Do I have to take part?
No. It is up to you to decide whether or not to take part. If you do decide to take part you will be asked to sign a consent form. If you decide to take part you are still free to withdraw at any time and without giving a reason. A decision to withdraw at any time, or a decision not to take part, will not affect the standard of care you receive.

What will happen to me if I take part? –
If you decide to take part you will attend a single out-patient visit at Charing Cross Hospital.

You will have specialized tests, which are carried out routinely in many people who come to our Center for a neuropathy screen.
These tests are:

1) Nerve conduction studies (NCS): it measures the degree of damage in the larger nerve fibres. During this test, a probe (electrode) electrically stimulates nerve fibres, which respond by generating their own electrical impulse: this is captured by another electrode placed along the nerve pathway or on the skin over a muscle.

2) Sympathetic skin response (SSR): it measures the function of sweating. During this test, the electrodes are placed on the skin of your hands or feet and will record the change in sweat activity following a stimulus.

3) Thermal threshold testing (TTT): it measures your ability to feel cold and warm sensation and mild pain sensation induced by cold and warmth. During this test, a probe will touch the skin on some part of your body (hands, feet) and you will be asked to press a button as soon as you feel cold and heat, cold pain and heat pain.

4) Electromyography (EMG): it studies the activity of muscles when they are at rest and when they contract. During this test, a fine needle is inserted into some muscles and picks up their activity.
   Insertion of a needle for the EMG test could be uncomfortable; however, if discomfort is felt, usually is mild and lasts only a couple of minutes. If you feel any unacceptable discomfort you can request that the procedure be stopped.

What do I have to do? –

You will need to attend a single outpatient visit at Charing Cross Hospital.

A doctor involved with this study, who performs these tests routinely, will carry-out the assessments described above.

What is the drug or procedure that is being tested? –

There are no drugs being tested in this study and no new treatments.

This study is simply looking at the functioning of your peripheral nerves. All the tests you will have are carried-out on a daily basis.

What are the side effects of any treatment received when taking part?

There are no additional treatments being tested in this study.

What are the possible disadvantages and risks of taking part?

The assessment techniques are entirely safe and non-invasive and there are no known risks from having these test performed.

Insertion of a needle for the EMG test could be uncomfortable; however, if discomfort is felt, usually is mild and lasts only a couple of minutes. If you feel any unacceptable discomfort you can request that the procedure be stopped.

All tests will be performed within your limits of tolerance.

Although pregnancy is not a contraindication, women who are pregnant will not be included.

What are the possible benefits of taking part? –

There are no clear immediate benefits of taking part. However, the information we get might help improve the future assessment and treatment of people born with thalidomide-induced limb malformation with regards to peripheral neuropathy symptoms.

What happens when the research study stops?

We will make any published results available to all participants in the study.

Contact details for further information:

If you are unsure about this study and would like to consider further before you make your decision, please take your time to do so. You may ask for further information by telephoning

The people to contact are :

Dr Omar Malik / Dr Richard Nicholas / Dr Alessia Nicotra
Charing Cross Hospital
West London Neurosciences Centre
Fulham Palace Road
London W6 8RP
Tel. 020 3311 1655

This completes Part 1 of the Information Sheet

If the information in Part 1 has interested you and you are considering participation, please continue to read the additional information in Part 2 before making any decision.

PART 2

What if relevant new information becomes available?

Sometimes during the course of a research project, new information becomes available about the technique that is being studied. If this happens, your research investigator will tell you about it and discuss whether you want to or should continue in the study. If you decide to continue in the study, you will be asked to sign an updated consent form.

What will happen if you don't want to carry on with the study?

You can withdraw from the study at anytime. If we collected data from you, we may still wish to use it in our analysis. Any information that can be clearly identified as yours can be destroyed if you wish.

What if there is a problem?

In case something goes wrong during your participation in the above research study, you will be covered by the standard NHS Hospital Indemnity for negligent or wrongful harm by Imperial College Healthcare NHS Trust.

Will my taking part in this study be kept confidential?

All information which is collected about you during the course of the research will be kept strictly confidential. Where applicable, any information about you which leaves the hospital will have your name and address removed so that you cannot be identified from it.

What will happen to the results of the research study?

The results of the study will be presented at scientific meetings and published in scientific journals. We can make any published results available to participants in the study by sending reprints by post or email upon request. Study participants will not be identifiable in any report or publication unless the participant has been specifically consented to release such information.

Who is organising and funding the research?

The study is organized by doctors at Charing Cross Hospital, Imperial College Healthcare Trust, London, in close collaboration with the Thalidomide Trust.

Who has reviewed the study? –

This study was approved by the East Central London Research Ethics Committee (REC) 1.

Contact details for further information:

You may ask for further information by telephoning

Dr Omar Malik / Dr Richard Nicholas / Dr Alessia Nicotra
Tel. 020 3311 1655

West London Neurosciences Centre
Imperial College HealthCare NHS Trust
Charing Cross Hospital
London, W6 8RF
Tel: +44 (0)20 3311 1655
Fax: +44 (0)20 3311 1300

Patient Identification Number for this study:

CONSENT FORM

(Version 4 – 21st December 2010)

Evaluation of the peripheral nervous system in patients with thalidomide-induced limb malformations

Investigators: Dr Omar Malik, Dr Richard Nicholas, Dr Alessia Nicotra

Please tick & initial box

1. I confirm that I have read and understand the information sheet dated 21st December 2010 (Version 4) for the above study and have had the opportunity to ask questions. ☐
2. I understand that my participation is voluntary and that I am free to withdraw at any time without giving any reason, and without my medical care or legal rights being affected. ☐
3. I understand that sections of any of my medical notes may be looked at by responsible individuals from Imperial College Healthcare NHS Trust or from regulatory authorities where it is relevant to my taking part in research. I give permission for these individuals to have access to my records. ☐
4. I agree to take part in the above study. ☐

______________________	______________	______________________
Name of subject	Date	Signature
______________________	______________	______________________
Name of person taking consent (if different from researcher)	Date	Signature
______________________	______________	______________________
Researcher	Date	Signature

Imperial College Healthcare NHS
NHS Trust

West London Neurosciences Centre
Imperial College HealthCare NHS Trust
Charing Cross Hospital
London, W6 8RF
Tel: +44 (0)20 3311 1655
Fax: +44 (0)20 3311 1300

Subject Identification Number for this study:

CONSENT FORM

(Version 4 – 21st December 2010)

Evaluation of the peripheral nervous system in patients with thalidomide-induced limb malformations

Investigators: Dr Omar Malik, Dr Richard Nicholas, Dr Alessia Nicotra

Please tick & initial box

1. I confirm that I have read and understand the information sheet dated 21st December 2010 (Version 4) for the above study and have had the opportunity to ask questions. ☐
2. I understand that my participation is voluntary and that I am free to withdraw at any time without giving any reason, and without my medical care or legal rights being affected. ☐
3. I understand that sections of any of my medical notes may be looked at by responsible individuals from Imperial College Healthcare NHS Trust or from regulatory authorities where it is relevant to my taking part in research. I give permission for these individuals to have access to my records. ☐
4. I agree to take part in the above study. ☐

______________________	______________	____________________
Name of subject	Date	Signature
______________________	______________	____________________
Name of person taking consent (if different from researcher)	Date	Signature
______________________	______________	____________________
Researcher	Date	Signature

The Thalidomide Trust

Registered Charity No. 266220

Trustees:
Sir Michael Wright
C.D. Lever
Professor O. Eremin
R.H. Lawson
M. Napier CBE QC
Professor C. Glendinning
J.S. Curtis
M. Winfield OBE
J.H. Howard
Professor Dame Lesley Southgate

Director:
Dr. M.W. Johnson BD PhD MSc

Registered Address:
1 Eaton Court Road
Colmworth Business Park
Eaton Socon
St Neots
Cambridgeshire
PE19 8ER

Telephone: 01480 474074
Fax: 01480 226777
E-mail: administration@thalidomidetrust.org
Web site: www.thalidomidetrust.org

Version 2

Dear Dr …

We understand that you are looking after one of the Beneficiaries of the Thalidomide Trust.

We are very pleased to inform you that we have established contact with Neurologists, Dr Omar Malik and Dr Richard Nicholas, Consultant Neurologists, based at the West London Neuro Sciences Centre, Charing Cross Hospital, who are particularly interested in the health issues and problems that are affecting many of the Beneficiaries. We understand that your patient Mr/Mrs/Ms (Insert name) is having significant health problems with particular clinical features and would welcome help with these symptoms. Drs Malik/Nicholas have agreed to see Beneficiaries if they are referred by their GP / PCT and we believe that a better understanding may be of benefit. Drs Malik/Nicholas already have some experience in this area. We believe that the best approach to the health problems that are affecting Beneficiaries is to build up the necessary experience in one centre, the resultant knowledge and expertise acquired can then be used in more effective management for other such patients, in the future.

The investigations and treatment for your patient's health problems should be provided by the NHS. The Thalidomide Trust is not able to pay for such treatment. However, the Thalidomide Trust has

agreed to pay for some specific tests that would not normally be done by the NHS, in order to establish the possible reasons for the health problems that your patient is experiencing. The Thalidomide Trust will pay for the patient travel expenses.

We hope that the results of the investigations that will be carried out may lead to better treatments being offered in the future to this group of patients. These tests will be done as part of a clinical research study and the full criteria and arrangements for this will be provided to you in other documents in due course.
We are very grateful to you for your support in this matter.

Yours sincerely,

On behalf of the Thalidomide Trust
